# Phylogeography and ecological niche modeling reveal evolutionary history of *Leiolepis ocellata* (Squamata, Leiolepidae)

**DOI:** 10.1002/ece3.7186

**Published:** 2021-01-20

**Authors:** Pattarapon Promnun, Nontivich Tandavanitj, Chalita Kongrit, Kritsayam Kongsatree, Phinit Kongpraphan, Wuttipong Dongkumfu, Detanan Kumsuan, Jenjit Khudamrongsawat

**Affiliations:** ^1^ Animal Systematics and Molecular Ecology Laboratory Department of Biology Faculty of Science Mahidol University Ratchathewi, Bangkok Thailand; ^2^ Department of Biology Faculty of Science Chulalongkorn University Pathumwan, Bangkok Thailand; ^3^ Doi Suthep‐Pui National Park Suthep, Mueang Chiang Mai District, Chiang Mai Thailand; ^4^ Doi Pha Klong National Park Ta Pha Mok, Long District Thailand; ^5^ Mae Puem National Park Mae Chai, Mae Chai District Thailand; ^6^ Mae Wa National Park Mae Wa, Thoen District, Lampang Thailand

**Keywords:** ecological niche modeling, *Leiolepis ocellata*, phylogeography, Thailand

## Abstract

*Leiolepis ocellata* is a lizard species distributing in topographically diverse habitats in northern Thailand. To explore its evolutionary history, 113 samples of *L. ocellata* were collected from 11 localities covering its distributional range in northern Thailand, and sequenced for mtDNA fragments (Cyt *b* and *ND2*). Pairwise comparisons across sampling localities yielded significant genetic differentiation (*F*_ST_ and Jost's *D*) but no clear pattern of isolation by distance could be demonstrated based on the Mantel test. Phylogenetic and network analyses highlighted six haplogroups. Their divergence times were estimated to occur during the Pleistocene, much more recent than major orogenic events affecting northern Thailand. Instead, the results suggested that lineage divergences, of particularly eastern and western haplogroups of the region, coincided with the major rivers in the region (Yom river and Ping river, respectively), indicating vicariance in response to riverine barriers. Furthermore, ecological niche modeling suggested an expansion of suitable habitats of *L. ocellata*, when LGM‐liked conditions. This expansion potentially facilitated their dispersal among adjacent localities leading to lineage diversification and genetic admixture, after the riverine divergence.

## Graphical abstract

*Leiolepis ocellata*, a lizard species distributed in topographically diverse habitats in northern Thailand, revealed 6 haplogroups. Lineage divergences, of particularly eastern and western haplogroups of the region, coincided with the major rivers in the region (Yom river and Ping river, respectively), indicating vicariance in response to riverine barriers.

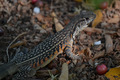

## 1 INTRODUCTION

Phylogeographic patterns of animal populations are influenced by major drivers, especially geographic barriers and the dispersal ability of organisms (Avise, 2009; Frankham et al., 2010; Moritz et al., 2000; Orsini et al., 2013; Pyron & Burbrink, 2010; Wiley, 1988). Geographic barriers form unsuitable conditions for species, limiting gene flow among populations and further genetic divergence (Wiley, 1988). Paleogeographic events, such as the formation of mountain ranges and the changing courses of rivers, are widely recognized as important processes in the evolutionary history of terrestrial animals (Bain & Hurley, 2011; Geissler et al., 2015; Gonçalves et al., 2018; Guo et al., 2011; Klabacka et al., 2020; Lin et al., 2010; Rakotoarisoa et al., 2013; Smissen et al., 2013). Other environmental factors especially climatic conditions are also important in influencing the dispersal ability of animals, shaping their geographic distributions, and contemporary population structures (Orsini et al., 2013; Pyron & Burbrink, 2010). Incorporating genetic data and species distribution modeling based on paleoclimatic information provides important insights into the historical dispersal patterns of species (Canestrelli et al., 2007; Kim et al., 2020; Moore, 1995; Ujvari et al., 2008; Werneck et al., 2012). The paleoclimatic events during the Pleistocene (2.5 mya–11.7 ka) are interesting since the multiple oscillations of climate, as glacial and interglacial periods, established repeated contraction and expansion of suitable habitat and species distribution range, contributing genetic divergence, and genetic admixture due to a subsequent secondary contact (Canestrelli et al., 2007; Ding et al., 2011; Graham et al., 2013; Grismer et al., 2014; Huang et al., 2013; Lanier & Olson, 2013; Lin et al., 2010; Nicolas et al., 2018).

The Indo‐Burma region is an intriguing biological hotspot for phylogeographic studies due to great biodiversity and complex landscapes and climates that are consequences of multiple paleogeographic events (Hall, 2009; Myers et al., 2000). That part in northern Thailand represents an important ecoregion topographically characterized by north‐south‐oriented mountain ranges and intermontane lowlands. The mountain ranges are extensions of the Tibetan Plateau, resulting from the accretion of the Indian subcontinent with Eurasia during the Tertiary (~50 mya; Tapponnier et al., 2001; Yin & Harrison, 2000). The Tibetan plateau is also the origin of large waterways in northern Thailand, including the Salween and Mekong rivers (Brookfield, 1998; Clark et al., 2004; Horton et al., 2002; Nie et al., 2018). Geological and biological evidences indicate that these two rivers formed the Siam river system, flowing via the present‐day northern Thai Ping river (the longest river in northern Thailand; Woodruff, 2010), and Yom river (Brookfield, 1998), respectively, into the Gulf of Thailand via the Chao Phraya river valley of central Thailand, during the Pleistocene (Hutchison, 1989; Woodruff, 2010). These historical events not only shaped the geological landscape but also brought a unique climate to northern Thailand promoting habitat heterogeneity (Heaney, 1991; Penny, 2001; White et al., 2004; Woodruff, 2010). These habitats harbor many endemic species and their conservation is of major concern (Bain & Hurley, 2011; Chan‐ard et al., 2015; Das, 2010; Trisurat et al., 2015). Despite evolutionary impact of these major historical, geographic, and climatic events, their influence on the phylogeography and population structure of terrestrial fauna confined to northern Thailand has scarcely been investigated.

Lizards of the genus *Leiolepis*, known as butterfly lizards, are diurnal and widely distributed in the Indo‐Burma region (Arunyavalai, 2003; Chan‐ard et al., 2015; Das, 2010; Grismer et al., 2014; Grismer & Grismer, 2010; Lin et al., 2010; Peters, 1971). *Leiolepis* originated on the Southeast Asian tectonic plate, which separated from the northern margin of the Australia‐New Guinea plate and collided with Asia around 120 mya or earlier (Macey et al., 2000). Nine species are recognized with the eyed butterfly lizard (*Leiolepis ocellata*) restricted to northern Thailand (Arunyavalai, 2003; Grismer et al., 2014; Peters, 1971; Promnun et al., 2020). To date, only limited studies have been conducted on its basic biology and genetics (Arunyavalai, 2003; Grismer et al., 2014; Grismer & Grismer, 2010; Peters, 1971; Promnun et al., 2020). *L. ocellata* is reported in open and dry intermontane lowlands (e.g., dry deciduous forest, grassland, and agricultural areas), throughout northern Thailand (Arunyavalai, 2003; Grismer et al., 2014; Peters, 1971; Promnun et al., 2020). Considering its distribution range in geologically complex region, *L. ocellata* populations provide opportunity for testing the roles of montane and riverine vicariance on the distribution and dispersal of terrestrial lizard in northern Thailand.

In this study, we sampled and sequenced *L. ocellata* across their geographic ranges in northern Thailand and conducted phylogenetic, population genetic analyses using two partial sequences of mitochondrial genes (Cyt *b* and *ND2*), and ecological modeling. The main goals of this study were to explore how paleogeographic events, including orogenic development and river systems, and paleoclimate might explain observed phylogeographic and population structure of *L. ocellata* populations in northern Thailand.

## 2 MATERIALS AND METHODS

### 2.1 Sample collection

**FIGURE 1:**
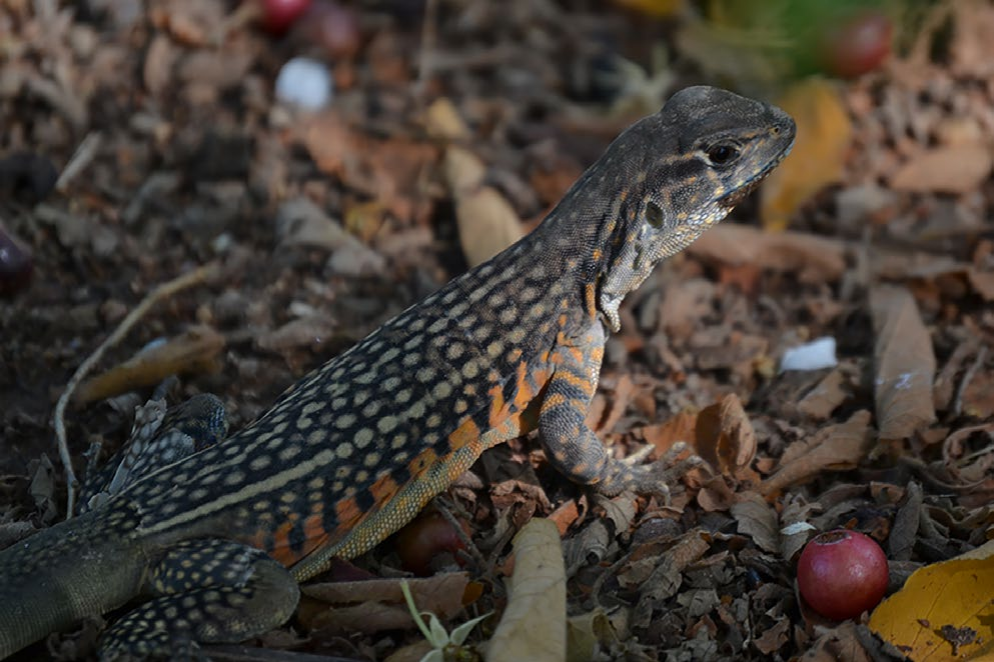
An individual of *Leiolepis ocellata* from northern Thailand. The photograph was taken by Pattarapon Promnun

**FIGURE 2:**
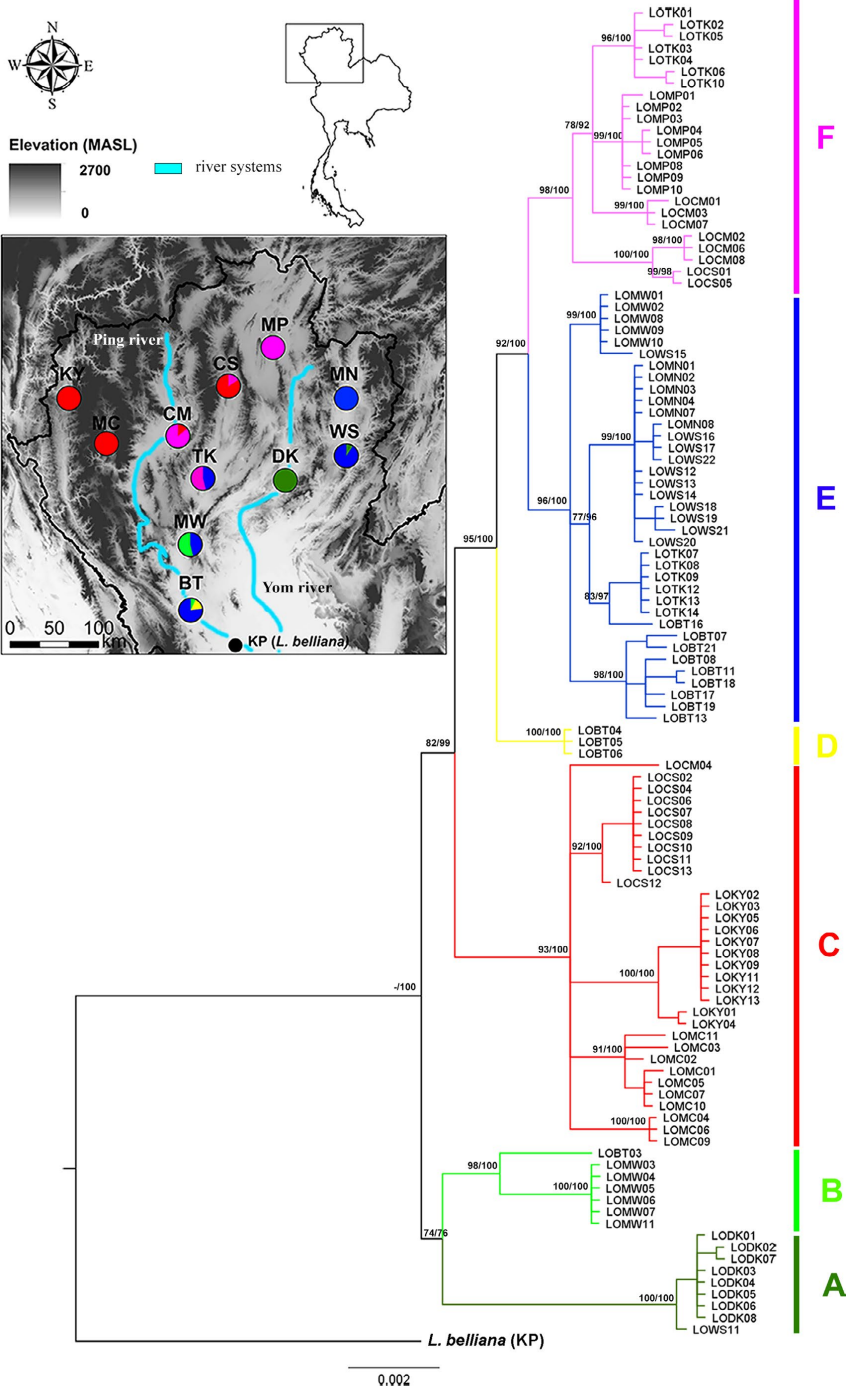
Bayesian Inference phylogenetic trees based on concatenated mtDNA (Cyt *b* and *ND2*) of *L. ocellata* with *L. belliana* from KP as an outgroup. The two last alphabets indicated code of sampling location following Table 1 (e.g., LOBT = Ban Tak). Numbers on each node were statistical supports of bootstrap values and posterior probabilities, respectively. The colors in map showed proportion of haplogroups in each sampling locality

In this study, *L. ocellata* were sampled and identified to species based on morphological characteristics (Arunyavalai, 2003; Peters, 1971). A total of 113 *L. ocellata* individuals (Figure 1) were collected from 11 localities during 2017–2019 (Table 1, Figure 2). These sampling localities covered most of the known distribution range of *L. ocellata* based on the range described by Grismer et al. (2014), specimen records from the Natural History Museum of Thailand (National Science Museum; THNHM), reports of the Department of National Parks, Wildlife and Plant Conservation (DNP), the Forest Industry Organization (FIO), local people, and our additional surveys, throughout northern Thailand (Promnun et al., 2020). Additionally, we sampled two individuals of *L. belliana* from Kamphaeng Phet Province (KP) to use as outgroups. The lizards were randomly captured by hand and traps. Tissues of tail tip (<1 cm in length) were clipped using sterile scissors. Collected tissues were preserved in 95% ethanol and stored at −20°C until further analyses.

**TABLE 1:** Sampling localities, sample size (*N*), number of haplotypes (*n*), haplotype diversity (*h*), nucleotide diversity (*π*) based on concatenated mtDNA

Code	Sampling localities	*N*	*n*	*h*	*π* (%)
BT	Ban Tak	13	10	0.95	0.59
CM	Mueang Chiang Mai	7	4	0.81	0.73
CS	Chae Son	12	3	0.44	0.42
DK	Doi Pha Klong	8	2	0.43	0.02
KY	Khun Yuam	12	2	0.30	0.06
MC	Mae Chaem	10	6	0.87	0.32
MN	Mueang Nan	6	2	0.33	0.01
MP	Mae Puem	9	3	0.64	0.03
MW	Mae Wa	11	2	0.55	0.67
TK	Thung Kwian	13	4	0.74	0.44
WS	Wiang Sa	12	6	0.85	0.37
All		113	42	0.97	1.08

### 2.2 Laboratory protocols

Genomic DNA was extracted using NucleoSpin tissue kit (Macherey‐Nagel). We amplified two mitochondrial regions using combinations of our designed primers CytbL (5′‐CCAACCAAGACCTTTGATCTG‐3′) and CytbH (5′‐AAGTATCCGGGTTGCATTTG‐3′) for partial cytochrome *b* (Cyt *b*) and ND2L1 (5′‐CCAAAGATGGGCTTGATTGT‐3′) and ND2H1 5′‐AAGTATCCGGGTTGCATTTG‐3′) for partial nicotinamide adenine dinucleotide dehydrogenase 2 (*ND2*). Polymerase chain reaction (PCR) was performed using AccuStart II GelTrack PCR SuperMix (Quanta BioSciences). Concentration of the PCR ingredients followed the suggested protocol from the manufacturer. The reactions were executed with the following steps: 95°C for 2 min followed by 32 cycles of 95°C for 35 s, annealing temperature for 35 s (60°C for Cyt *b* and 55°C for *ND2*), 72°C for 1 min using Eppendorf Mastercycler gradient thermocycler. Products were visualized with 1.5% agarose gel electrophoresis and purified using NucleoSpin Gel and PCR Clean‐up (Macherey‐Nagel). The PCR products were sent for sequence analyses with the Applied Biosystems BigDye Terminator v3.1 Cycle Sequencing Kit following the protocol from the manufacturer.

### 2.3 Population genetic analyses

Sequences were aligned using ClustalW in MEGA7 v. 7.0.21 (Kumar et al., 2016) and deposited in GenBank (accession no. MN728559‐MN728676 and MN734580‐MN734692). For each locality of *L. ocellata*, genetic diversity indices, including number of haplotypes (*n*), haplotype diversity (*h*), and nucleotide diversity (*π*), were calculated using DnaSP5 (Librado & Rozas, 2009). Based on sampling localities, pairwise *F*_ST_ was calculated using Arlequin v.3.5.2.2 (Excoffier & Lischer, 2010) to assess population genetic differentiation, with 10,000 permutations and performing Bonferroni correction for *p* values. Since the *F* statistics may provide underestimated results for markers with high polymorphism (Jost, 2008), pairwise Jost's *D* was also calculated using GenAIEx v6.5 (by setting the haplotype data as homozygote genotype data; Peakall & Smouse, 2012), with 9,999 permutations and performing Bonferroni correction for *p* values. To further test the correlation between genetic distance and geographic distance among populations, isolation by distance (IBD; Wright, 1943) was tested using using Mantel test, implemented in RStudio v1.3 (RStudio team, 2020), “vegan” package, with 999 permutations (Dixon, 2003). Pairwise *F*_ST_ calculated by Arlequin was used as a genetic distance matrix. Geographic distances among populations were measured from the distance between sampling localities using QGIS (QGIS Development Team, 2020).

### 2.4 Phylogenetic and network analyses

Phylogenetic trees of *L. ocellata* collected in this study were constructed based on concatenated mtDNA (Cyt *b* and *ND2*) haplotypes under Maximum Likelihood (ML) and Bayesian Inference (BI) approaches with *L. belliana* collected from Kamphaeng Phet Province (KP), serving as an outgroup. Dataset of mtDNA sequences were partitioned allowing the evolutionary model to specify each partition separately. Kakusan4 (Tanabe, 2007) was used to select the best‐fit evolutionary model under Akaike information criterion (AIC; Akaike, 1974) and Bayesian information criterion (BIC; Schwarz, 1978) for ML and BI, respectively. The selected models for the ML trees were: Cyt *b*, J1 Invariant; and *ND2*, TN93 Gamma; and for the BI trees were: Cyt *b*, HKY85 Invariant; and *ND2*, HKY85 Gamma. The ML trees were constructed using RAxML v8.2.12 (Stamatakis, 2014) on CIPRES (Miller et al., 2010) with 1,000 pseudoreplicates to estimate branch confidence values. The bootstrap value of 70% or higher was considered as significant support (Huelsenbeck & Hillis, 1993). The BI trees were constructed in MrBayes v3.2.6 (Huelsenbeck & Ronquist, 2001) under a Metropolis‐coupled, Markov chain Monte Carlo (MC‐MCMC) approach, started from random tree, run twice in parallel with a four‐chain analysis for 10 million generations. The trees were sampled every 100 generations and 25% of the generations were discarded as burn‐in. The outputs were checked by examining Effective Sample Size (ESS; >200) in Tracer v1.7.1 (Rambaut et al., 2018). The remaining trees were used to estimate the consensus topology, branch length, and posterior probability. The posterior probability of 95% or higher was considered as a significant support (Larget & Simon, 1999). The trees were visualized and edited in FigTree v1.4.3 (Rambaut, 2009). We additionally established Median‐Joining Network for mtDNA dataset to illustrate relationships among haplotypes using Popart (Leigh & Bryand, 2015).

### 2.5 Divergence time estimation

Divergence times were estimated from the Cyt *b* dataset of *L. ocellata* and *L. belliana*. Analyses were conducted in BEAST v1.10.4 (Drummond & Rambaut, 2007) using uncorrelated relaxed clock model with constant‐size coalescent prior and the model of nucleotide substitution described above. Since recent fossil calibration for *Leiolepis* was not available, we used the substitution rates of Cyt *b* derived from of many species of lizards following previous study of *L. reevesii* (Lin et al., 2010). We assumed mean substitution rate as a normal distribution, with a mean of 0.01 and a standard deviation of 0.005 substitution per site per million years. MCMC ran for 50 million generations, sampled every 100 generations, with 10% were discarded as burn‐in. The outputs were checked by examining Effective Sample Size (ESS; >200) in Tracer v1.7.1 (Rambaut et al., 2018) and summarized in TreeAnnotator v1.10.4 (Drummond & Rambaut, 2007).

### 2.6 Ecological niche modeling

Ecological niche modeling was conducted using Maxent v3.4.1 (Phillips et al., 2006) to investigate the effects of past and present climatic conditions on populations of *L. ocellata*. The Maxent predicted habitat suitability map, based on presence data of the species of interest and environmental layers. Coordinates for sites where *L. ocellata* was present were obtained from our previous surveys (Promnun et al., 2020; museum records were excluded) and additional surveys during 2018–2020. The coordinates located within the same grid on environmental layers were considered the same point and 41 points were obtained. Nineteen bioclimatic variables layers (Table S1) at three epochs, Last Glacial Maximum (LGM; CCSM and MIROC datasets), Holocene (CCSM and MIROC datasets), and the present time (monthly average conditions for 1970–2000) were downloaded from the WorldClim database (www.worldclim.org) at a resolution of 30 s (~1 km^2^; Hijmans et al., 2005). As the preliminary models predicted that the distribution of *L. ocellata* occurred in northern Thailand, we clipped and only presented the map of northern Thailand using QGIS. To select the most important predictor variables, we ran initial models with the default setting for each period. We selected the predictor variables from the layer of the present time, which were >10% for percent contribution and permutation importance, and less correlated to each other (Pearson correlation coefficient |*r*| < 0.80; Khanum et al., 2013). The predictor variables included bio3, bio7, and bio15. The subsequent models were generated with a randomly selected 75% and the remaining 25% of presence data being used as training and testing data, respectively, for model validation (Corbalán et al., 2011). Models were under 10 replications of bootstrap replicated run type (5,000 iterations) and other parameters with default setting. The areas under the curve (AUC) of a receiver operating characteristics (ROC) plot were considered to evaluate performance of models. The higher the AUC values appear, the more reliable the models are (Eskildsen et al., 2013). The contribution of each selected variable was assessed from the percent contribution and permutation importance.

## 3 RESULTS

### 3.1 Population genetic analyses

The final alignments of concatenated partial mitochondrial DNA from 113 *L. ocellata* individuals revealed 1953 bp (142 variable sites, 113 parsimony informative sites), which contained 1,071 bp of Cyt *b* (79 variable sites, 56 parsimony informative sites) and 882 bp of *ND2* (63 variable sites, 57 parsimony informative sites), and defined 42 haplotypes. Genetic diversity among sampling localities was shown in Table 1. Number of haplotypes (*n*) ranged from 2 to 10, haplotype diversity (*h*) ranged from 0.30 to 0.95, and nucleotide diversity (*π*) ranged from 0.01% to 0.73%, for all localities. The overall genetic diversity *h* and *π* were 0.97 and 1.08%, respectively.

Pairwise *F*_ST_ and Jost's *D* among all sampling localities were used to indicate genetic differentiation. Pairwise *F*_ST_ was significant (*p* < .05) for most comparisons, except for localities MN‐WS, while Jost's *D* was significant (*p* < .05) for most comparisons, except for localities MN‐MW and MN‐WS (Table 2). There were three localities, DK, KY, and MC, those relatively had the higher degree of genetic differentiation for both *F*_ST_ and Jost's *D* when paired with other localities. The analysis of isolation by distance based on Mantel test showed low and non‐significant correlation between genetic distance and geographic distance matrices (*r* = 0.2, *p* > .05).

**TABLE 2:** Genetic differentiation between sampling localities for *F*_ST_ (below diagonal) and Jost's *D* (above diagonal). Values with bold were statistically supported by *p* < .05 after performing Bonferroni correction

	BT	CM	CS	DK	KY	MC	MN	MP	MW	TK	WS
BT	–	**0.07**	**0.13**	**0.24**	**0.22**	**0.16**	**0.06**	**0.09**	**0.06**	**0.05**	**0.05**
CM	**0.36**	–	**0.10**	**0.27**	**0.21**	**0.15**	**0.10**	**0.04**	**0.09**	**0.04**	**0.09**
CS	**0.57**	**0.50**	–	**0.29**	**0.11**	**0.05**	**0.16**	**0.15**	**0.13**	**0.13**	**0.15**
DK	**0.78**	**0.81**	**0.86**	–	**0.35**	**0.30**	**0.28**	**0.29**	**0.22**	**0.27**	**0.23**
KY	**0.78**	**0.79**	**0.72**	**0.98**	–	**0.11**	**0.25**	**0.24**	**0.22**	**0.23**	**0.24**
MC	**0.65**	**0.63**	**0.44**	**0.90**	**0.77**	–	**0.19**	**0.19**	**0.16**	**0.17**	**0.18**
MN	**0.42**	**0.57**	**0.75**	**0.99**	**0.97**	**0.84**	–	**0.12**	0.10	**0.06**	<0.01
MP	**0.57**	**0.39**	**0.76**	**0.98**	**0.97**	**0.85**	**0.96**	–	**0.11**	**0.05**	**0.10**
MW	**0.34**	**0.41**	**0.56**	**0.75**	**0.77**	**0.62**	**0.53**	**0.61**	–	**0.08**	**0.08**
TK	**0.33**	**0.28**	**0.62**	**0.84**	**0.83**	**0.70**	**0.49**	**0.47**	**0.43**	–	**0.05**
WS	**0.33**	**0.48**	**0.67**	**0.84**	**0.86**	**0.73**	<0.01	**0.71**	**0.44**	**0.38**	–

### 3.2 Phylogenetic and network analyses

As concatenated mtDNA trees based on both BI and ML approaches provided similar topology, only the BI tree was shown in Figure 2. The phylogenetic tree topology strongly supported the monophyly of *L. ocellata* in northern Thailand (100% posterior probability) when *L. belliana* from KP was treated as an outgroup. Within the *L. ocellata* group, six haplogroups, A‐F, were identified. Haplogroup A (100% bootstrap value and 100% posterior probability) consisted of all *L. ocellata* from DK and one sample from WS. Haplogroup B (98% bootstrap value and 100% posterior probability) was closely related to haplogroup A and consisted of individuals from the southernmost localities, MW and BT. Haplogroup C (93.7% bootstrap value and 100% posterior probability) contained all samples from KY and MC, in the west of the region, with many samples from CS and some samples from CM. Haplogroup D (100% bootstrap value and 100% posterior probability) consisted of three samples from BT. Haplogroup E (100% bootstrap value and 100% posterior probability) consisted of samples from five localities in the east and south of northern Thailand (MN, WS, TK, MW, and BT). Finally, haplogroup F (98% bootstrap value and 100% posterior probability) was distributed in central northern Thailand, and found in MP, CS, CM, and TK.

**FIGURE 3:**
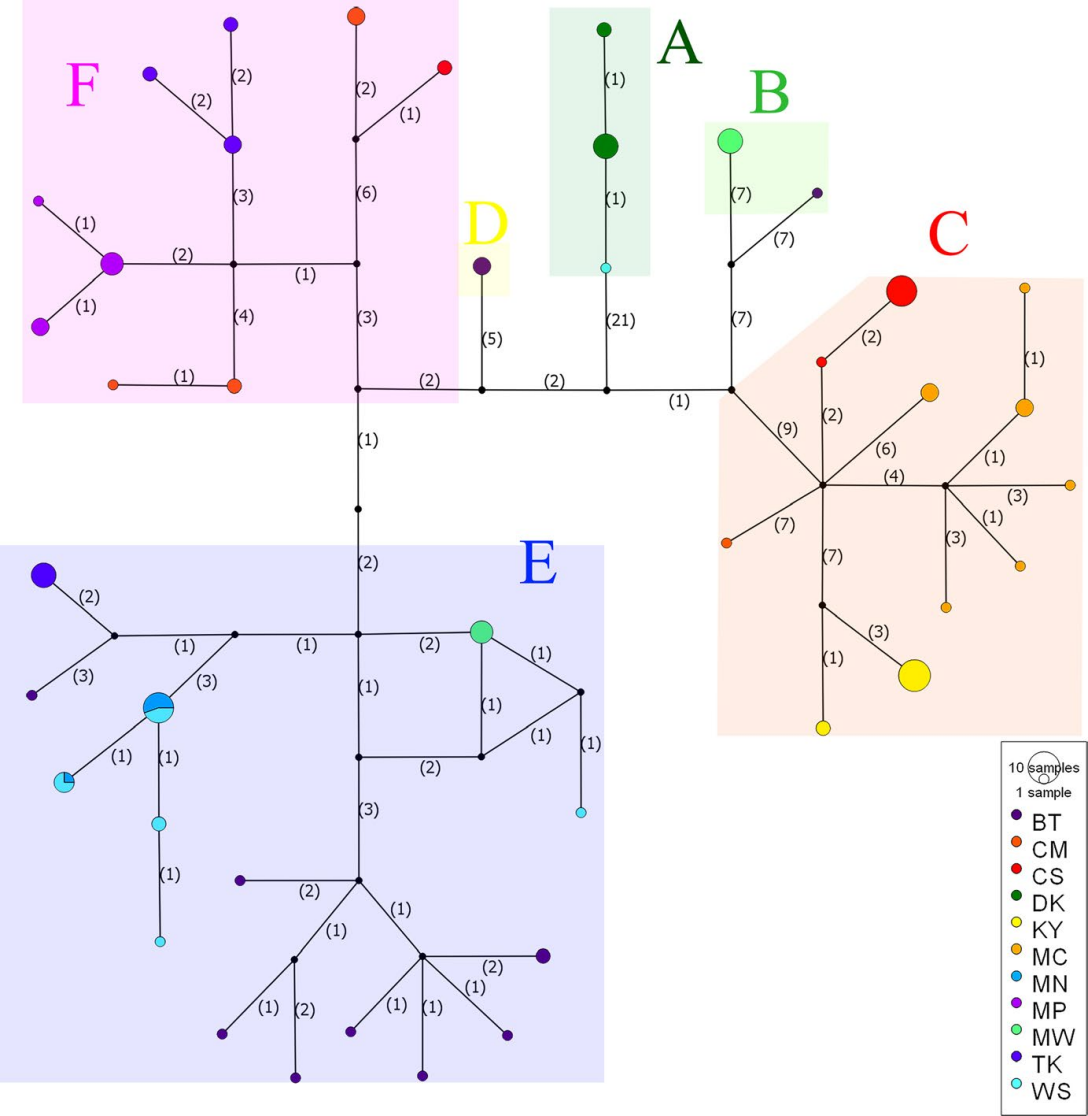
Network analysis of 42 haplotypes of *L. ocellata* based on concatenated mitochondrial genes (Cyt *b* and *ND2*)

The network analyses of concatenated mitochondrial genes provided similar results to those of the phylogenetic trees but revealed slightly different genealogy (Figure 3). A total 42 haplotypes of *L. ocellata* formed six recognizable groups (A‐F) identified in the trees. Each locality harbored private haplotypes and most haplotypes were unique to their geographic localities except for two haplotypes shared between MN and WS.

### 3.3 Divergence time

**FIGURE 4:**
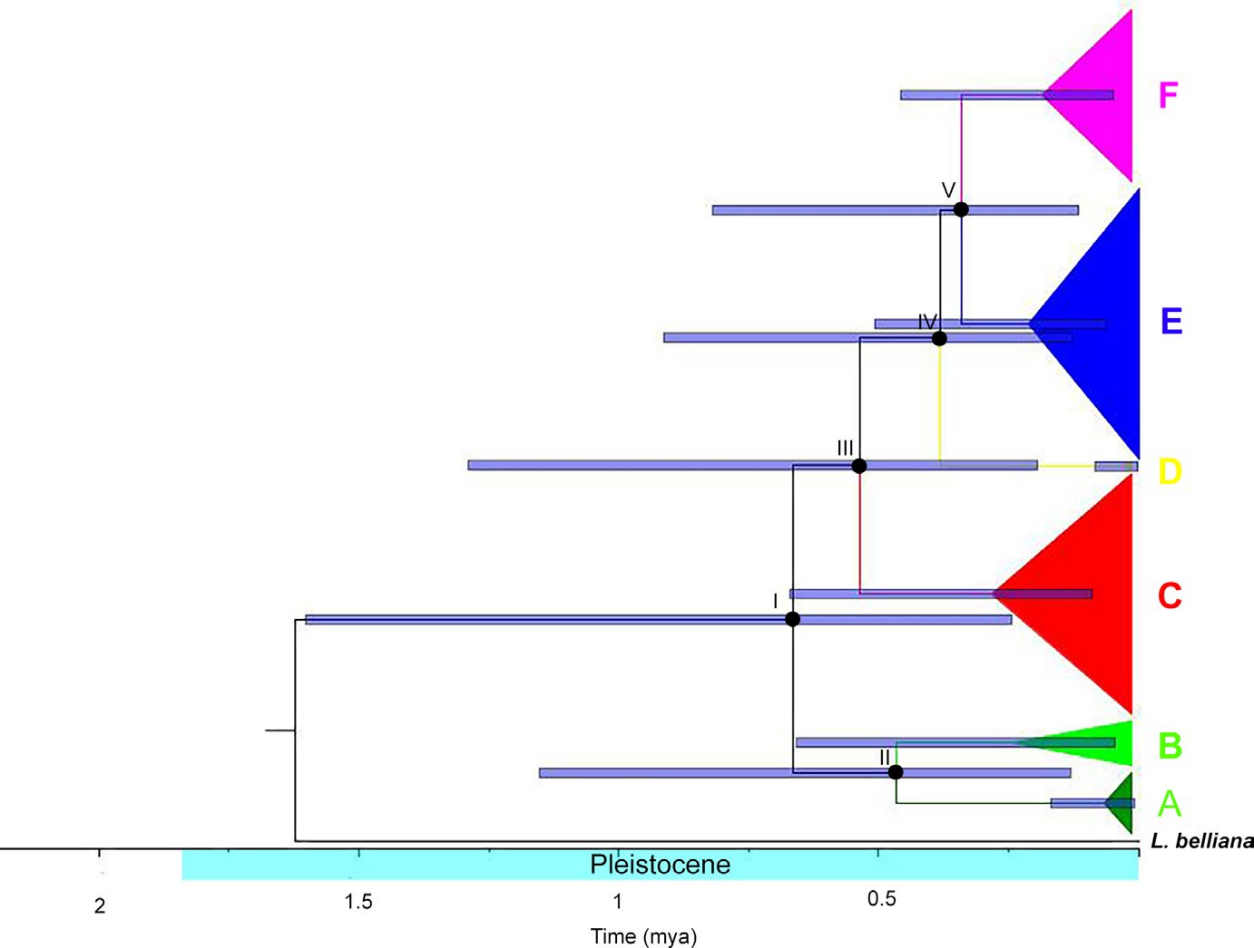
Bayesian Inference (BI) tree based on Cyt *b* showed divergence time estimation of *L. ocellata* and *L. belliana* serving as an outgroup, using uncorrelated lognormal relaxed clock in BEAST v1.10.4. Filled circles with roman numbers indicated nodes of major divergences (details were in Table 3). Blue bars represented the 95% HPD (Bayesian credible interval) for timing of divergence

Based on Cyt *b* mtDNA sequences, the coalescence times of the *L. ocellata* haplogroups were all estimated to have occurred during the Pleistocene (Table 3, Figure 4). The first divergence of *L. ocellata* (node I in Figure 4) was estimated to at 0.81 mya (HPD = 0.24–1.62). Divergence within the first group (node II in Figure 4) occurred at 0.61 mya (HPD = 0.14–1.29). Within the second group, divergence between haplogroup C and the remaining haplogroups (node III in Figure 4) was estimated at 0.69 mya (HPD = 0.20–1.40). Haplogroup D was further diverged from E and F group (node IV in Figure 4) at 0.46 mya (HPD = 0.13–0.93). Divergence between the last two haplogroups, E and *F* (node V in Figure 4), occurred at 0.40 mya (HPD = 0.12–0.82).

**TABLE 3:** Estimated divergence times since the most recent common ancestor (tMRCA) with 95% highest posterior density interval (HPD) for six haplogroups of *L. ocellata*. Time scale in million years ago (mya)

Node	Group	tMRCA (95%HPD)
I	All *L. ocellata* (A‐F)	0.81 (0.24–1.62)
II	A and B	0.61 (0.14–1.29)
III	C and group of D, E, and F	0.69 (0.20–1.4)
IV	D and group of E and F	0.46 (0.13–0.93)
V	E and F	0.40 (0.12–0.82)

### 3.4 Species distribution modeling

**FIGURE 5:**
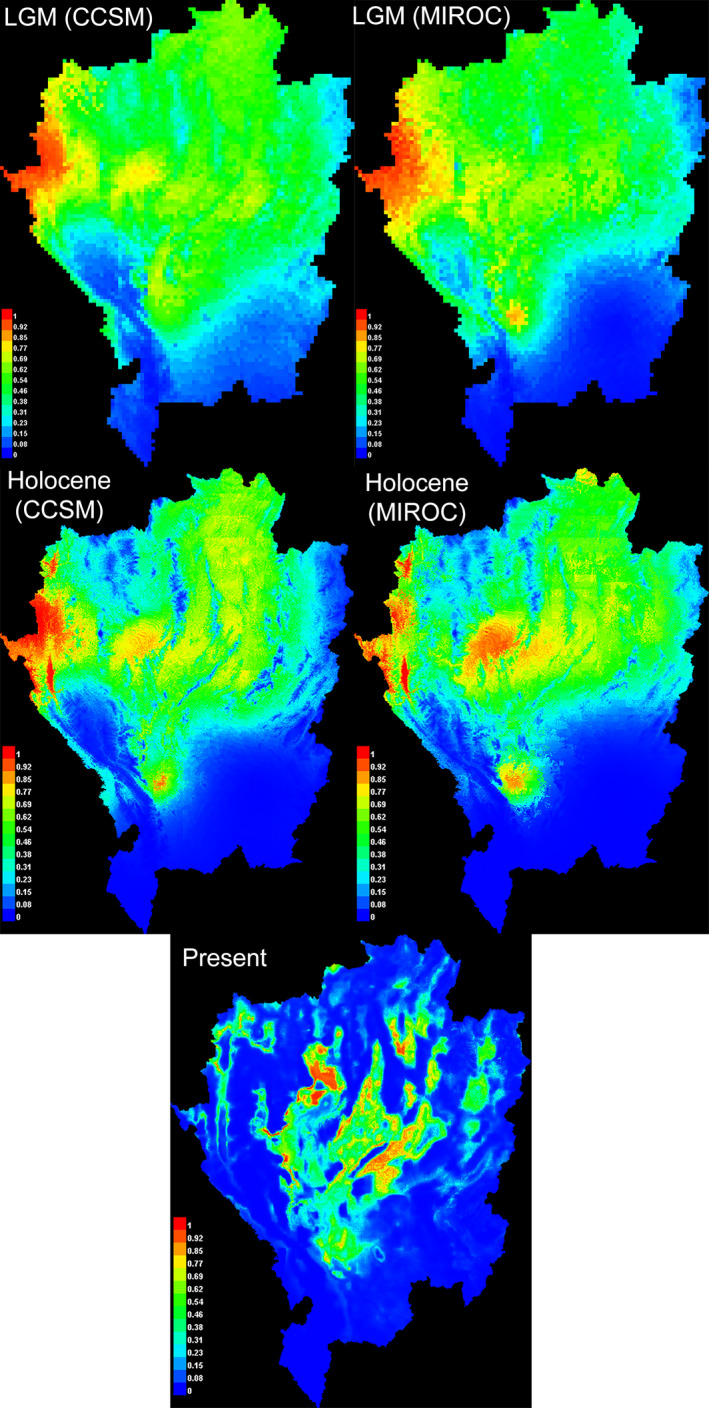
Predicted distribution of *L. ocellata* based on LGM, Holocene, and Present bioclimatic data. The highest probability of occurrence was shown in red color, while the lowest probability of occurrence was shown in blue color

Maxent predicted the distributions of *L. ocellata* during three epochs, LGM, Holocene, and at the present time, based on selected predictor variables (Figure 5). The AUC values for all of these models were > 0.8, indicating the robustness and reliability of their predictions. During the LGM and Holocene, the predicted distribution ranges of *L. ocellata* were similar under CCSM and MIROC environmental layers. *L. ocellata* had the largest distribution range throughout northern Thailand during the LGM, extending as far south as Ban Tak (BT). Those ranges were slightly contracted during the Holocene. Low habitat suitability occurring in the Holocene appeared in the north of the Thanon Thong Chai range and upper course of the Ping River. At the present time, the distribution ranges were further contracted and mostly restricted to the central parts of the region. While the unsuitable habitats along the Thanon Thong Chai range and Ping river further isolated western populations (KY and MC), other unsuitable habitats along the Yom river in the east partially isolated eastern populations (DK, WS, and MN).

## 4 DISCUSSION

### 4.1 Population genetics of *L. ocellata*

Exploring the phylogeography and population genetics of organisms provides valuable insights for both evolutionary processes and conservation of biodiversity (Avise, 2009; Frankham et al., 2010; Manel et al., 2003). The effects of paleogeographic and climatic events on terrestrial animals were widely observed in topographically diverse regions, which often serve as biological hotspots (e.g., Carnaval et al., 2009; Grismer et al., 2014; Guo et al., 2011; Lin et al., 2010; Zhu et al., 2016). Northern Thailand constitutes part of the Indo‐Burma biodiversity hotspot, possessing complex geography and high habitat heterogeneity, but has rarely been considered for phylogeographic study. Our investigation on *L. ocellata* may be the first phylogeographic and population genetic study of any terrestrial fauna confined to northern Thailand.

Evidence from mtDNA enabled us to uncover significant genetic diversity within *L. ocellata* as indicated by its high haplotype diversity (*h*) and nucleotide diversity (*π*). Based on Cyt *b*, the values of these diversity indices of *L. ocellata* (*h* = 0.95 and *π* = 0.98) were comparable to those observed in *L. reevesii* (Hainan populations: *h* = 0.97 and *π* = 1.00 and Guangxi and Guangdong populations: *h* = 0.94 and *π* = 1.06; Lin et al., 2010). However, the genetic diversity within some populations such as those at DK and MN was relatively low, which could be due to many factors, such as overexploitation as food by local people that caused population decline and reduction in genetic diversity (Arunyavalai, 2003; Lin et al., 2010).

Analyses from the concatenated mtDNA implied strong population genetic structure of *L. ocellata* populations as indicated by significant genetic differentiation (pairwise *F*st and Jost's *D*) and lack of shared haplotypes among populations other than for the MN‐WS. This population structure was likely a consequence of low dispersal ability of lizards (Clark et al., 1999; Dubey & Shine, 2010; Koumoundouros et al., 2009; Orsini et al., 2013). Dispersal of *Leiolepis* seems to be limited by their behaviors and life history (Arunyavalai, 2003). Based on our field observations, each burrow of the ground‐dwelling *Leiolepis* was usually occupied by one adult (with juveniles in the same burrow for mature females). Juveniles usually dispersed to adjacent areas after maturation and remained in a particular burrow unless they were disturbed. It was also noticed that some adults protected their burrows and adjacent areas against other individuals indicating that territorial behavior that may exist in *Leiolepis*. Such habits of *L. ocellata* together with other geographical factors might further attribute to its phylogeographic structure at a regional scale.

### 4.2 Phylogeographic pattern and divergence between major lineages

Both phylogenetic and network analyses, based on concatenated mtDNA, provided a similar phylogeographic pattern within *L. ocellata* in which six haplogroups could be identified. To further infer the potential factors related to this phylogeographic pattern, divergence times were estimated from Cyt *b* tree using substitution rates from other reptiles (Agamidae), following the study of *L. reevesii* (Lin et al., 2010). Without fossil calibration of *Leiolepis*, the use of these substitution rates should be interpreted with caution. According to these rates, the divergence times of six major lineages of *L. ocellata* were all estimated to occur during the Pleistocene. These divergence times were congruent with those of other species, which were suggested to be influenced by several scenarios (e.g., Canestrelli et al., 2007; Gonçalves et al., 2018; Graham et al., 2013; Grismer et al., 2014; Lin et al., 2010; Nicolas et al., 2018). While in general, population genetic structure may be explained by an isolation by distance pattern (IBD; Orsini et al., 2013; Wright, 1943), where genetic distance increases with increasing geographic distance, but this scenario did not explain *L. ocellata* population structure as the correlation between genetic and geographic distances was not significant.

Vicariance, the process by which historical gene flow is disrupted by physical barriers such as mountain ranges, is one of the leading hypotheses to explain current distribution and phylogeographic structure of terrestrial animals (Brown et al., 2002; Pyron & Burbrink, 2010; Wiley, 1988). As *L. ocellata* were mostly confined to lowlands among mountain ranges in northern Thailand, we first considered that mountain ranges may play a significant role in their population structuring. However, this hypothesis was rejected due to the fact that the same haplogroups were observed on different sides of the mountains. For example, haplogroup C was present on both western (KY and MC), and eastern (CM, and CS) sides of the highest mountain of Thailand, Thanon Thong Chai range. Furthermore, the Pleistocene divergence time was much later than the time of orogenic events in northern Thailand, which took place during the Tertiary (Tapponnier et al., 2001; Yin & Harrison, 2000). This pattern was different from that of *L. reevesii* populations on Hainan Island, where genetic differentiation occurred due to the presence of geographic barrier, the Wushishan mountain range (Lin et al., 2010), though elevation of the Wushishan range (1,840 m) was lower than that of the maximum elevation of the Thanon Thong Chai range (2,526 m).

River systems are much more likely to have act as geographic barriers to terrestrial species in the Indo‐Burma region (Bain & Hurley, 2011; Geissler et al., 2015; Gonçalves et al., 2018; Klabacka et al., 2020; Rakotoarisoa et al., 2013). The rivers in northern Thailand, like the mountains, are oriented north‐south. As we have indicated, the Salween in the west and the Mekong in the east joined and flowed to the Siam river, presently recognized as the Chao Phraya river, during the Pleistocene (Hutchison, 1989; Woodruff, 2010). The capture of these river systems would have increased water volume such that they presented geographic barriers influencing the phylogeographic pattern of *L. ocellata* in northern Thailand (Klabacka et al., 2020). The Mekong river was thought to flow through the presently recognized Yom river (Brookfield, 1998) and may be responsible for the early divergence of *L. ocellata*. The ancestors of *Leiolepis* originated in the southern hemisphere and arrived in Southeast Asia approximately 120 mya (Macey et al., 2000), an ancestor of *L. ocellata* may have come from the south and distributed in southernmost lowlands in northern Thailand. The Yom river might have fragmented the ancestral *L. ocellata* into two major groups corresponding with node I in Figure 4. In eastern side of the lower Yom river, haplogroup A, principally located in DK, potentially diverged earlier, accounting for the relative high differentiation between the DK population and those of other localities. Part of the haplogroup A, population from DK, may have subsequently dispersed to adjacent localities such as WS, and also diverged as a closely related haplogroup B in MW (node II; Figure 4). The Salween river was hypothesized to flow through the presently recognized Ping river (Woodruff, 2010) in the west of northern Thailand. This may have isolated populations of *L. ocellata* found to the west of the Yom river into haplogroups C and D‐F, mostly distributed in the west and central of northern Thailand, respectively (node III; Figure 4). This riverine vicariance due to the Ping river was supported by relatively high genetic differentiation between the populations on the west bank (KY and MC) and those populations to the east.

The phylogeographic pattern of *L. ocellata* was explained both by vicariance and also by dispersal following riverine divergence, as indicated by ecological niche modeling. The models revealed that distribution range of *L. ocellata* was largest during the LGM. This was probably due to the fact that *L. ocellata* prefers dry habitat, which would have reached its maximum extent throughout northern Thailand at that time (Heaney, 1991; Penny, 2001). Suitable dry habitat then became reduced and contracted during the Holocene up to the present as it was replaced by moister tropical forest mosaic (Gathorne‐Hardy et al., 2002; Heaney, 1991). Considering climate oscillation during the Pleistocene, there may have been cycles in which drier habitats repeatedly expanded and contracted. The expansion of dry habitats under LGM‐liked conditions would likely have facilitated dispersal of *L. ocellata* among adjacent localities, where strong geographic barriers were absent. Such a dispersal scenario could explain the lineage diversification and admixture among haplogroups in the localities, located in the middle of the region between the Ping and Yom rivers (e.g., those at BT, MW, and TK). Moreover, dispersal of *L. ocellata* during the range expansions or minor changes in river course may have resulted in secondary contact between haplogroups confined to different banks of the rivers (Canestrelli et al., 2007; Ding et al., 2011; Graham et al., 2013). Localities CS and CM, for example, had an admixture of haplogroup C and F, which were found in the western and eastern banks of the Ping river, respectively. When the Salween river diverged from the Ping river, into its presently recognized course in Myanmar (Brookfield, 1998; Hutchison, 1989), the water volume of the Ping river was potentially reduced, especially in its upper reaches. This together with the expansion of dry habitats may have facilitated the dispersal of individuals of haplogroup C on the western bank of the Ping river (KY and MC) to the eastern (CS and CM). A similar divergence of the Mekong and Yom rivers might account for easterly dispersal of centrally distributed haplogroup E, to localities such as MN and WS (Brookfield, 1998; Hutchison, 1989). However, the presence of dominant haplogroup, instead of genetic admixture, in some localities (e.g., MN) may be a result of random effects such as genetic drift (Frankham, 1995). The hypothesis of recent dispersal after riverine divergence is also supported by relatively small genetic differentiation among localities, except for KY, MC, and DK that diverged earlier (Kim et al., 2020).

In conclusion, the lineage diversification among *L. ocellata* populations has taken place too recently to have been influenced by the orogeny of the mountain ranges, but coincided temporally and spatially with the development of riverine geographic barriers. These caused the early phylogenetic split of eastern and western populations. The original lineage diversification was then modified by subsequent dispersal and secondary contact followed by genetic admixture, especially in the central of northern Thailand. Our conclusions were derived from an integrative approach, combining population genetics, phylogeography, and ecological niche modeling, which enabled us to suggest hypotheses concerning the evolutionary history of *L. ocellata* in response to natural drivers in northern Thailand. We believed that further investigations of other species will assist clarification of phylogeographic puzzles in the northern Thailand biodiversity hotspot.

## CONFLICT OF INTEREST

We have no conflict of interest.

## AUTHOR CONTRIBUTION

**Pattarapon Promnun:** Conceptualization (equal); Data curation (lead); Formal analysis (lead); Investigation (lead); Methodology (equal); Project administration (lead); Validation (equal); Visualization (lead); Writing‐original draft (lead); Writing‐review & editing (equal). **Nontivich Tandavanitj:** Conceptualization (equal); Formal analysis (supporting); Methodology (equal); Supervision (equal); Validation (equal); Visualization (equal); Writing‐review & editing (equal). **Chalita Kongrit:** Conceptualization (equal); Formal analysis (supporting); Methodology (equal); Visualization (equal); Writing‐review & editing (equal). **Kritsayam Kongsatree:** Resources (equal). **Phinit Kongpraphan:** Resources (equal). **Wuttipong Dongkumfu:** Resources (equal). **Detanan Kumsuan:** Resources (equal). **Jenjit Khudamrongsawat:** Conceptualization (equal); Formal analysis (supporting); Funding acquisition (lead); Investigation (supporting); Methodology (equal); Supervision (equal); Validation (equal); Visualization (equal); Writing‐review & editing (equal).

## Supporting information


**Table S1**

**Table S2**

**Table S3**

## Data Availability

All sequencing data are available at the GenBank, accession no. MN728559‐MN728676 and MN734580‐MN734692. All climatic data were downloaded from www.worldclim.org (https://doi.org/10.1002/joc.5086).
