# Supplementary material for: Phylogeography and ecological niche modeling reveal evolutionary history of Leiolepis ocellata (Squamata, Leiolepidae)
Source: Ecol Evol. 2021 Jan 20;11(5):2221–33. doi: 10.1002/ece3.7186 (PMC7920770; doi:10.1002/ece3.7186)
Supplement: Supplementary file 1 — Table S1 [file ECE3-11-2221-s001.docx]

**Table S1.** 19 bioclimatic variables

|  | Name |
| --- | --- |
| bio1 | Annual mean temperature |
| bio2 | Mean diurnal range (mean of monthly (max temp – min temp)) |
| bio3 | Isothermality (bio2/bio7) (*100) |
| bio4 | Temperature seasonality (standard deviation *100) |
| bio5 | Max temperature of warmest month |
| bio6 | Min temperature of coldest month |
| bio7 | Temperature annual range (bio5-bio6) |
| bio8 | Mean temperature of wettest quarter |
| bio9 | Mean temperature of driest quarter |
| bio10 | Mean temperature of warmest quarter |
| bio11 | Mean temperature of coldest quarter |
| bio12 | Annual precipitation |
| bio13 | Precipitation of wettest month |
| bio14 | Precipitation of driest month |
| bio15 | Precipitation seasonality (coefficient of variation) |
| bio16 | Precipitation of wettest quarter |
| bio17 | Precipitation of driest quarter |
| bio18 | Precipitation of warmest quarter |
| bio19 | Precipitation of coldest quarter |
