# Supplementary material for: Phylogeography and ecological niche modeling reveal evolutionary history of Leiolepis ocellata (Squamata, Leiolepidae)
Source: Ecol Evol. 2021 Jan 20;11(5):2221–33. doi: 10.1002/ece3.7186 (PMC7920770; doi:10.1002/ece3.7186)
Supplement: Supplementary file 2 — Table S2 [file ECE3-11-2221-s002.docx]

**Table S2.** Genetic distance (km; above diagonal) and geographic distance (below diagonal) between sampling locations

|  | BT | CM | CS | DK | KY | MC | MN | MP | MW | TK | WS |
| --- | --- | --- | --- | --- | --- | --- | --- | --- | --- | --- | --- |
| BT | 0 | 0.01 | 0.01 | 0.02 | 0.02 | 0.01 | 0.01 | 0.01 | 0.01 | 0.01 | 0.01 |
| CM | 189.9 | 0 | 0.01 | 0.02 | 0.02 | 0.01 | 0.01 | 0.01 | 0.01 | 0.01 | 0.01 |
| CS | 202.3 | 60.3 | 0 | 0.02 | 0.01 | 0.01 | 0.01 | 0.01 | 0.01 | 0.01 | 0.01 |
| DK | 156.7 | 133.8 | 94.3 | 0 | 0.02 | 0.02 | 0.02 | 0.02 | 0.02 | 0.02 | 0.02 |
| KY | 232.2 | 107.9 | 167.9 | 231.9 | 0 | 0.01 | 0.02 | 0.01 | 0.02 | 0.02 | 0.02 |
| MC | 149.9 | 81.1 | 133.0 | 169.8 | 81.1 | 0 | 0.01 | 0.01 | 0.01 | 0.01 | 0.01 |
| MN | 277.0 | 191.7 | 131.4 | 120.4 | 299.3 | 259.1 | 0 | 0.01 | 0.01 | 0.01 | 0 |
| MP | 287.9 | 129.2 | 86.9 | 156.9 | 211.2 | 210.1 | 112.9 | 0 | 0.01 | 0.01 | 0.01 |
| MW | 55.9 | 150.4 | 151.6 | 101.2 | 207.6 | 130.9 | 221.4 | 235.4 | 0 | 0.01 | 0.01 |
| TK | 147.7 | 59.6 | 55.3 | 78.5 | 153.4 | 96.4 | 164.9 | 142.1 | 99.4 | 0 | 0.01 |
| WS | 257.2 | 188.2 | 128.7 | 100.9 | 296.1 | 250.4 | 24.7 | 126.8 | 202.0 | 154.5 | 0 |
