# Supplementary material for: Phylogeography and ecological niche modeling reveal evolutionary history of Leiolepis ocellata (Squamata, Leiolepidae)
Source: Ecol Evol. 2021 Jan 20;11(5):2221–33. doi: 10.1002/ece3.7186 (PMC7920770; doi:10.1002/ece3.7186)
Supplement: Supplementary file 3 — Table S3 [file ECE3-11-2221-s003.docx]

**Table S3.** Canonical coefficients of selected bioclimatic variables along axes

|  | Axis1 | Axis2 | Axis3 |
| --- | --- | --- | --- |
| bio2 | -0.248 | -0.117 | 0.147 |
| bio3 | -0.558 | -0.006 | -0.009 |
| bio5 | 0.198 | -0.331 | 0.173 |
| bio15 | 0.469 | 0.185 | -0.423 |
| bio19 | -0.463 | 0.241 | 0.321 |
